# Prevalence of idiopathic epilepsy and structural epilepsy in 74 Boxer dogs in a referral hospital

**DOI:** 10.3389/fvets.2022.956648

**Published:** 2022-08-19

**Authors:** Tina Loncarica, Federica Balducci, Marco Bernardini

**Affiliations:** ^1^Neurology Unit, Anicura Portoni Rossi Veterinary Hospital, Bologna, Italy; ^2^Department of Animal Medicine, Production and Health, Clinical section, University of Padua, Legnaro, Italy

**Keywords:** Boxer, etiology, seizure, dog, structural epilepsy, idiopathic epilepsy

## Abstract

The prevalence of idiopathic epilepsy and structural epilepsy in Boxer dogs is unknown. The aim of this retrospective study was to evaluate the prevalence of structural and idiopathic epilepsy in the Boxer population. A total of 74 Boxer dogs were included in the study from the database of one referral hospital and the following were recorded: signalment, history, clinical findings and results of advanced diagnostic imaging. Five dogs (6.8%) were diagnosed with idiopathic epilepsy, of which one was in the <6 months age group, three were in the 6–72 months age group and one was in the >72 months age group. Sixty-nine dogs (93.2%) were diagnosed with structural epilepsy. Sixty-six had a suspected intracranial neoplasia: Eight were in the 6–72 months age group and represent 66.7% of the dogs in that age group. The other fifty-eight were in the >72 months age group and represent 96.7% of the dogs in that age group. In our Boxer population, 81.8% of the patients had a suspected intra-axial tumor and 22.7% of dogs with an intracranial pathology nevertheless had a normal neurological examination. In conclusion, in the majority of boxer patients the cause of epilepsy is a suspected intracranial neoplasia regardless of the age at presentation. Considering the finding in this study of a low prevalence of presumed idiopathic epilepsy in the Boxer breed, it is recommended that patients who satisfy Tier I confidence level of the “International Veterinary Epilepsy Task Force” (IVETF) also undergo an MRI study of the brain.

## Introduction

Epilepsy is a common disorder in veterinary medicine and is defined as the predisposition to develop epileptic seizures (1). According to the International Veterinary Epilepsy Task Force (IVETF), epilepsy can be classified based on the underlying etiology into two categories: idiopathic or structural. Idiopathic epilepsy (IE) is diagnosed when a genetic background is identified or suspected, or an underlying cause has not been found (1). Numerous epidemiologic and genetic studies have been conducted to date, revealing a predisposition for genetic or suspected genetic epilepsy in Vizlas, Finnish Spitz, English Springer Spaniels, Belgian Tervurens, Belgian Sheepdogs, Bernese Mountain dogs, Labrador Retrievers, Lagotto Romagnolo, Border Collies and Irish Wolfhounds, among other breeds (2–13). Structural epilepsy (SE) is characterized by the presence of epileptic seizures caused by an intracranial pathology (vascular, inflammatory/infectious, traumatic, congenital, neoplastic or degenerative anomaly), confirmed through an MRI study of the brain, examination of the cerebrospinal fluid, genetic tests or necropsy (1).

One recent study evaluated the prevalence of IE and SE in a large general population of dogs undergoing MRI for epileptic seizures; it found that 53.8% of dogs had IE and 45.1% had SE (14). 75.5% of the patients aged between 7 and 72 months were diagnosed with IE and 3% were diagnosed with intracranial neoplasia. In the >72 months age group, 34.1% of dogs were diagnosed with IE and 63,9% with SE, of which 43.2% had intracranial neoplasia (14). Another study conducted in a referral center found that 52.1% of dogs were affected by SE, 31.2% of them due to a neoplastic condition (15).

Two recent studies identified the Boxer as the breed with the highest prevalence of seizures (16, 17). In addition, a predisposition for Boxer dogs to develop intracranial tumors, both intra-axial and extra-axial, is reported (15, 17–24), as well as a predisposition for hereditary epilepsy (25).

There are currently no data regarding the prevalence of IE and SE in Boxer dogs. The aim of this study was to provide estimates of the prevalence of IE and SE in a population of Boxers that presented to our hospital and underwent MRI for epileptic seizures.

## Materials and methods

Data were retrospectively collected between February 2007 and December 2020 from the database of the AniCura I Portoni Rossi Veterinary Hospital by searching for boxer dogs and then using the following keywords: “seizure(s),” “epileptic seizure(s)” or “epilepsy.” Only dogs that underwent MRI of the brain were included in the study. Dogs were excluded if the medical records were incomplete; if the records were diagnostic or suggestive for a metabolic/toxic pathology; or if the MRI study was not available for re-examination. The medical records were reviewed and the following data were recorded: signalment, clinical history, results of physical and neurological examination, hematology and serum biochemistry results, age at the first seizure, MRI findings, and cerebrospinal fluid (CSF) analysis. Dogs were divided into categories based on the age of the first seizure: ≤6 months, 7–72 months, and >72 months, following a published paper (14). All cases were managed by a board-certified neurologist or a resident in neurology under supervision. The neurological examination was defined as normal, abnormal, or not evaluable (because of a recent seizure). The MRI was performed with a 0.22 T (MrJ, Paramed Health Service, Genua, Italy) from February 2007 to March 2018 and with a 1.5 T (Vantage Elan, Canon Medical Systems Europe, The Netherlands) from April 2018 to December 2020. The acquired sequences had to include at least: sagittal and transverse T2-weighted; transverse FLAIR; and transverse T1-weighted before and after the administration of a gadolinium-based contrast medium. The MRI studies were then reviewed by a board-certified neurologist to determine whether seizures were due to structural pathology. Based on the history, the neurological examination, the MRI results, and the CSF analysis, each dog was categorized as affected by IE or one of the following structural conditions: congenital malformation, inflammatory disease, vasculopathy, degenerative disease, trauma and neoplasm. Within the neoplastic group, patients were further divided into two groups based on the intra- or extra-axial localization of the mass lesion.

Descriptive statistics were performed utilizing Microsoft Excel for Mac. Mean was used for all descriptive statistics: gender, age at first seizure, neurological examination (altered, normal or not evaluable), MRI findings (altered or normal), etiology, age at which the patient was subjected to MRI, and localization of the neoplastic lesion (extra-axial or intra-axial).

## Results

One thousand and seventy-two boxers presented to our hospital during the study period, 368 (34.3%) of which had a neurological examination for a suspected neurological disease and 91 (8.5%) had seizures. Of these 91 patients, 16 were excluded due to the lack of an MRI study and 1 because of reactive seizures. Thus, 74 patients (6.9%) were included in the study.

Two out of 74 cases were <6-months old. There was a 6-month old female patient diagnosed with IE and a 5-month old male patient diagnosed with a meningoencephalocele (MEC).

Twelve out of 74 patients (16.2%) were aged between 7 and 72 months. Five were males (41.7%) and 7 females (58.3%). The mean age of presentation of the first epileptic seizure was 44.8 months and the mean age at which they were subjected to advanced diagnostic procedures was 49.3 months. Eight out of 12 patients (66.7%) had an MRI study showing a presumptive neoplastic lesion (2 extra-axial, 1 suspected extra-axial, and 5 intra-axial). The mean age of these patients was 59 months. Of these eight patients, 2 (25.0%) had a normal and 6 (75%) had an abnormal neurological examination. Three out of 12 patients (25.0%) had a normal neurological examination with a normal MRI study and a normal cerebrospinal fluid examination (performed in only one out of 3 patients). In these cases, the suspected diagnosis was IE. The mean age of these patients was 19.3 months. The remaining dog in this age group was 7-months old and the MRI was suggestive of an ischemic event.

Sixty out of 74 patients (81.1%) were older than 72 months. Twenty-nine were male (48.3%) and 31 were female (51.7%). The mean age of presentation of epileptic seizures was 107.7 months and the mean age at which they were subjected to advanced diagnostics was 109.1 months. Fifty-eight of 60 patients (96.7%) had an MRI study suggestive of a neoplastic condition. The mean age of these patients was 108.4 months. Of these 58 dogs, 13 (22.4%) had a normal neurological examination and 38 (65.5%) had an abnormal neurological examination. In 7 dogs (12.1%) the neurological examination was either not performed because of the recent administration of antiepileptic drugs, or was not reliable because of a recent seizure. One out of 60 patients (1.7%) had a suspected diagnosis of IE. The age of this patient was 98 months. One out of 60 patients (1.7%), aged 74 months, had an MRI suggestive of an ischemic event.

Overall, the majority of patients (89.2%) were diagnosed with suspected neoplastic disease. Of this group, 12.1% were aged between 7 and 72 months and 87.9% were older than 72 months. We found a total of 54 intra-axial neoplasms and 12 extra-axial neoplasms. Eleven dogs underwent a histological examination, which confirmed the neoplastic nature of the lesions. There were 6 meningiomas, 1 glioma, 1 ependymoma, 1 leptomeningeal lymphomatosis and 2 metastases (1 hemangiosarcoma and 1 carcinoma). IE accounted for 5 out of 74 cases (6.8%), of which 3 (60%) were aged between 7 and 72 months, 1 (20%) was younger than 6 months and 1 was over 72 months. Two out of 74 patients (2.7%) had a suspected vascular disease, one of which was aged between 7 and 72 months and the other was older than 72 months. One out of 74 patients (1.4%) had a diagnosis of congenital disease, at <6 months old.

## Discussion

This study analyzes the causes of epilepsy in the Boxer dogs. The motivation behind the study is the authors' impression that this breed presents epidemiologic and etiologic peculiarities among dogs affected by epileptic seizures.

Across the 14 year study period, of all the Boxers presented to our hospital 34.3% had a neurological examination for a suspected neurological disease. A previous study, conducted in Switzerland, found that 33% of general canine population had a presumptive neurological diagnosis (26). This data was in agreement with the results of our study, but it is possible that within this large group the pathologies were distributed in a different way according to the breed and the age range taken into consideration. In fact, when focusing on epilepsy, our data differed from that present in the literature. In our population of Boxer dogs fulfilling the inclusion criteria, the prevalence of epileptic seizures was found to be 6.9%. However, of the 17 patients that were excluded, only 1 was diagnosed with reactive seizures. In the remaining 16, clinical history and blood work were not suggestive of a metabolic condition, so either IE or SE would be a likely diagnosis. The real prevalence of epileptic seizures in our population of Boxer dogs might consequently be higher, close to 8.4% (90/1,072). The prevalence of epileptic Boxers in a referral institution has not been investigated to date. Two studies conducted on the prevalence of epileptic seizures in the general canine population in primary veterinary care reported a percentage of 0.75–0.82% and identified the Boxer as the breed with the highest seizure prevalence (1.8–2.3%, respectively) (16, 17).

In our population there were only two puppies aged between 0 and 6 months. In a paper on dogs aged <1 year, the mean age at the first seizure in dogs with IE was 6.8 months, while in dogs with SE it was 7.5 months, and in dogs experiencing reactive seizures it was 4 months (27). As such, one plausible reason for the low prevalence of epileptic dogs aged <6 months is that reactive seizures could be more common in this age group. This low prevalence strictly overlaps that found in another study, where 20 of 900 dogs were aged <6 months at the time of their first seizure (14). In the same study, 70% of the dogs (14/20) were diagnosed with SE, mostly due to congenital malformations, and 30% with IE (14).

The main causes of epileptic seizures in pediatric patients are congenital malformations and inflammatory conditions (28). The Boxer is not among the breeds with the highest incidence of the most common congenital conditions, such as hydrocephalus and lissencephaly (28, 29). The puppy with SE in our data set had a MEC, a sporadically occurring disease potentially underdiagnosed because diagnosis requires advanced imaging (30). Seizures are the main presenting neurologic sign in dogs with MEC (30). In our dog, MRI showed an intranasal MEC with robust meningeal post-contrast enhancement, possibly due to inflammation. Infiltration of inflammatory cells contributing to abnormal excitability of cortical neurons is one of the pathogenetic mechanisms hypothesized to explain seizures in patients with MEC (31).

The lack of pediatric patients with an inflammatory condition in our study is likely due to several causes. Autoimmune encephalitis is uncommon at this age (28, 32) and Boxers are not at a higher risk of this kind of inflammation (32). Infectious encephalitis was regarded as a frequent cause of seizures in dogs in the recent past and Canine Distemper virus was the most common form (33, 34). Although vaccination for Canine Distemper virus does not ensure a total immunity against the neurological form of the disease and not all dogs are vaccinated, in recent decades its use has become so widespread that there has been a severe reduction in the occurrence of CDV-related seizures.

A previous study reported that IE was the final diagnosis in 75.5% of cases in a canine population affected by epileptic seizures, aged between 6 months and 6 years (14). In the same study, neoplasia was diagnosed or suspected in only 3% of dogs. These data differ considerably from those found in our study, where 66.7% of Boxers had an MRI suggestive of brain neoplasia and only 25% had a diagnosis of IE. Among the dogs diagnosed with or suspected of having a brain tumor, one quarter showed a normal neurological examination. The average age of dogs in this age group with suspected intracranial neoplasia was 59 months.

In a recent paper, the predictive value of age at the time of the first epileptic seizure to differentiate between IE and SE was investigated combining and analyzing the data from two previously published studies (1, 35). It found that a 6-year cut off was a better predictor than a 5-year cut off to determine the likelihood of a dog being affected by IE. We have not performed statistical analysis on our dataset. However, based on our data it seems reasonable that in Boxers a 6-year cut off is too high and we recommend performing an MRI of the brain, after the exclusion of reactive seizures, in Boxers 5 years old or even younger, independent of the results of the neurological examination.

With regard to the age group over 72 months, we found that 96.7% of our Boxers had a diagnosis of epilepsy due to a histologically confirmed or an MRI suspected neoplasia, and only 1.7% had a diagnosis of IE. In a published study on patients over 6 years of age, intracranial neoplasia was diagnosed in 43.2% of the cases and was the primary differential diagnosis for this age group (14). A similar study investigated the etiology of epilepsy in dogs 5 years of age and older and found that 49.5% of patients had a diagnosis of intracranial neoplasia (36). If we set the lower age limit of our population at 5 years, patients with a confirmed or suspected diagnosis of intracranial neoplasia equaled 96.9% (63 out of 65). In other words, lowering the age limit to 5 years did not cause any change in the percentage of epileptic Boxers in this age group who had intracranial neoplasia.

According to our data, regardless of the age of the first seizure, Boxers have a 93.2% chance to suffer from SE and a 90.4% chance of being affected by a suspected or confirmed neoplastic condition. A recent study investigated the prevalence of IE and SE in the canine population, where it emerged that the Boxer is the fourth most common breed to have structural pathologies underlying the development of epileptic seizures (14).

These data may reflect the high predisposition of Boxer dogs for developing brain neoplasia compared to the general population (15, 17–24). However, it is the authors' opinion that other causes have to be hypothesized to justify such a high prevalence. The first factor could be related to the referral nature of our hospital. It is likely that many of these dogs have been initially investigated by their referring veterinarians, that no clinically significant abnormalities on blood or urinalysis were found, and it was decided to either evaluate the response to an antiepileptic treatment or to wait and observe the frequency and the severity of the seizures. In this way, treatment-responders, i.e., dogs with mild neurological impairment and dogs with low frequency seizures, were not referred, generating a severe bias in our dataset. A second factor which again relates to the referral nature of our hospital could lead to an opposite clinical situation; for example, cases showing a very quick clinical evolution toward either cluster seizures / status epilepticus or severe neurological deficits may actually not be referred. A severe clinical scenario may discourage owners from pursuing a final diagnosis through often expensive investigations. Vascular and inflammatory conditions and, in some instances, IE could constitute the cause of such severe clinical conditions (37–39) and, again, bias the overall prevalence when not investigated.

Two recent studies, which considered dogs that have undergone intracranial surgery for tumor removal, highlighted the Boxer as one of the most affected breeds, representing 12.9–18.2% of patients (23, 24). Eighty-five percent of the Boxers had an intra-axial tumor (20). In our study, 81.8% of Boxers with an MRI-suspected neoplasia had an intra-axial lesion and 18.2% had an extra-axial lesion. This finding agrees with a previous study, where Boxers are the most represented breed amongst patients with intra-axial tumors (14, 20). In an older study it was hypothesized that the predisposition of the ancestral bulldog, from which the Boxer breed derives, to certain diseases (chemodectomas, proliferation of fibrillar astrocytes) is caused by the altered anatomy of the respiratory tract. This alteration leads to prolonged hypoxia, which in humans can stimulate the development of fibrillar gliosis (22). The same study also argues that some brachycephalic breeds (English bulldog, Boxer and Boston terrier) are more likely than other brachycephalic breeds to develop glial tumors. The neurological examination is usually expected to be abnormal in dogs with intracranial disease, yet it is known that a normal neurological examination does not exclude a structural brain lesion. In a previous study, 23% of dogs <6 years old at the onset of seizures and 22% of dogs over 6 years old at the onset of seizures had a normal neurological examination with abnormal MRI results (40). In our Boxer population we found similar results, since 22.7% of patients with an intracranial neoplasia had a normal neurological examination. In light of this evidence, we recommend performing an MRI in epileptic Boxers in spite of a normal neurological examination, especially if they experience the first epileptic seizure in adulthood.

In the Boxer dog a genetic predisposition to IE has been proposed, on the basis of a high mortality rate in seizuring subjects of 4 years of age or less (25). No diagnostic procedures were performed in that study to rule out SE and support this hypothesis. Indeed, the Boxer is not listed among the breeds for which IE has a demonstrated or supposed predisposition. Evidence to support a predisposition for IE was not found in our study. Only 6.8% of patients were diagnosed with IE out of the entire Boxer population, regardless of age, and 25% of patients in the range between 6 months and 6 years. However, if we lower the upper age limit of this group to 5 years based on the previously discussed prevalence of tumors, the proportion of IE rises to 47%, approximating but not equaling what is found in the general canine population in the same age group (14). Again, it is likely that subjects with good control of epileptic seizures after IVETF Tier 1 protocols will not be referred to specialist centers for a complete diagnostic workup and this may lead to an underestimation of the real incidence of IE in Boxers. However, this may not be sufficient to explain the low level of IE seen here in Boxers; our institution sees many dogs of other breeds referred for refractory epilepsy and so it is possible that Boxers are less affected by this condition. Further studies are needed to determine the real prevalence of IE in Boxers.

Our study has several limitations, mainly related to its retrospective nature. In the 14-year span many veterinarians performed the neurological examination and subtle abnormalities may have been overlooked in some cases. However, neurological examinations were always performed by experienced clinicians or residents under strict supervision by ECVN diplomates, thus minimizing this situation. Due to the emergency conditions in which some cases are referred, many patients were examined following an epileptic seizure or status epilepticus. Therefore, the percentage of patients with SE and an abnormal neurological examination may have been overestimated. These dogs may have had a normal neurological examination if they had been examined at a time distant from the seizure episode. This limitation may negatively bias our percentage of dogs with a normal neurological examination despite SE, but emphasizes the need to perform advanced imaging in Boxers with a history of seizures. Other limitations of the study are the variable MRI protocols and different MRI scanners used, due to the long study period. The majority of the studies were performed with a low-field MRI scanner. Because of the lack of a histopathological confirmation in most cases, it cannot be excluded that inflammatory or vascular conditions could have been misdiagnosed for a low-grade, non-contrast-enhancing glioma in a few cases.

In conclusion, Boxer dogs experiencing seizures have a high probability of having intracranial neoplasia, regardless of their age and the result of the neurological examination. It is therefore always advisable to perform advanced imaging to better define the diagnosis and hence an adequate prognosis.

## Data availability statement

The raw data supporting the conclusions of this article will be made available by the authors, without undue reservation.

## Ethics statement

Ethical review and approval was not required for the animal study because this is a retrospective clinical study. Written informed consent for participation was not obtained from the owners because for the retrospective nature and for the long study period (14 years).

## Author contributions

TL and FB were responsible for the study conception. TL collected the data. FB and MB supervised data analysis and manuscript editing. TL and MB wrote the manuscript. All authors contributed to the article and approved the submitted version.

## Conflict of interest

The authors declare that the research was conducted in the absence of any commercial or financial relationships that could be construed as a potential conflict of interest.

## Publisher's note

All claims expressed in this article are solely those of the authors and do not necessarily represent those of their affiliated organizations, or those of the publisher, the editors and the reviewers. Any product that may be evaluated in this article, or claim that may be made by its manufacturer, is not guaranteed or endorsed by the publisher.
